# Obesity-related indices are associated with albuminuria and advanced kidney disease in type 2 diabetes mellitus

**DOI:** 10.1080/0886022X.2021.1969247

**Published:** 2021-08-30

**Authors:** Yu-Lun Ou, Mei-Yueh Lee, I-Ting Lin, Wei-Lun Wen, Wei-Hao Hsu, Szu-Chia Chen

**Affiliations:** a Division of Endocrinology and Metabolism, Department of Internal Medicine, Kaohsiung Medical University Hospital, Kaohsiung Medical University, Kaohsiung, Taiwan; b Faculty of Medicine, College of Medicine, Kaohsiung Medical University, Kaohsiung, Taiwan; c Department of Internal Medicine, Kaohsiung Municipal Siaogang Hospital, Kaohsiung Medical University, Kaohsiung, Taiwan; d Division of Nephrology, Department of Internal Medicine, Kaohsiung Medical University Hospital, Kaohsiung Medical University, Kaohsiung, Taiwan

**Keywords:** Obesity-related index, type 2 diabetes mellitus, albuminuria, advanced kidney disease

## Abstract

Obesity is an important risk factor for the development of diseases including diabetes, hypertension, and cardiovascular disease. However, few reports have investigated the relationships between these obesity-related indices and diabetic nephropathy. The aim of this study was to evaluate associations between obesity-related markers with albuminuria and advanced kidney disease in patients with type 2 diabetes mellitus (DM). Obesity-related indices including body mass index (BMI), waist-to-hip ratio (WHR), waist-to-height ratio (WHtR), body roundness index (BRI), conicity index (CI), lipid accumulation product (LAP), visceral adiposity index (VAI), body adiposity index (BAI), abdominal volume index (AVI), body shape index (BSI), and triglyceride glucose (TyG) index were measured. Albuminuria was defined as a urine albumin/creatinine ratio of ≥30 mg/g. Advanced kidney disease was defined as an estimated glomerular filtration rate (eGFR) <30 ml/min/1.73 m^2^. A total of 1872 patients with type 2 DM (mean age 64.0 ± 11.3 years, 809 males and 1063 females) were enrolled. In multivariable analysis, 11 high obesity-related indices (BMI, WHR, WHtR, LAP, BRI, CI, VAI, BAI, AVI, ABSI, and TyG index) were significantly associated with albuminuria. In addition, high BMI, WHR, WHtR, LAP, BRI, CI, VAI, and AVI were significantly associated with eGFR <30 ml/min/1.73 m^2^. The results of this study showed that various obesity-related indices were significantly associated with albuminuria and advanced kidney disease in patients with type 2 DM. Screening may be considered in public health programs to recognize and take appropriate steps to prevent subsequent complications.

## Introduction

Chronic kidney disease (CKD) is an increasing global public health problem due to the high risk of progression to end-stage renal disease (ESRD) and associated morbidity and mortality [1]. Taiwan has been reported to have the highest prevalence and incidence rates of ESRD requiring renal replacement therapy globally [2]. Diabetic nephropathy is a complication of diabetes mellitus (DM) characterized by high levels of urine albumin excretion, loss of glomerular filtration rate (GFR), and diabetic glomerular lesions. Diabetic nephropathy is the most common cause of CKD, ultimately leading to ESRD and the need for renal replacement therapy [2]. Patients with diabetic nephropathy have higher risks of ESRD, cardiovascular disease (CVD) and even death compared to diabetic patients without kidney disease [3]. Therefore, identifying the risk factors for diabetic nephropathy is of great importance.

Obesity is also a global public health problem with an increasing prevalence [4]. Obesity has been shown to markedly increase the risk of developing diseases including metabolic syndrome, diabetes, hypertension, and CVD [5]. It is worth noting that the prevalence of obesity in CKD patients is quite high. For example, in the United States, it increased from 38.1% between 1999 and 2002 to 44.1% between 2011 and 2014, while in Ireland, it was approximately 35.3% [6,7]. Various obesity-related indices are used to evaluate obesity, including body mass index (BMI), waist-to-hip ratio (WHR), waist-to-height ratio (WHtR), lipid accumulation product (LAP) [8], body roundness index (BRI) [9], conicity index (CI) [10], visceral adiposity index (VAI) [11], body adiposity index (BAI) [12], abdominal volume index (AVI) [13], body shape index (ABSI) [14], and triglyceride glucose (TyG) index [15]. Previous studies have reported an association between high BMI and the risk of ESRD in the general population [16,17]. Other studies have reported that a combination of BMI and WHtR may have the best clinical utility to identify adult patients at risk of CVD [18,19], and BRI and WHtR have also been reported to be strongly associated with nonalcoholic fatty liver disease [20]. However, few reports have investigated the relationships between these obesity-related indices and diabetic nephropathy.

Therefore, in this study, we collected the data of more than 1800 patients with type 2 DM in southern Taiwan to explore associations between obesity-related indices (BMI, WHR, WHtR, LAP, BRI, CI, VAI, BAI, AVI, ABSI, and TyG index) with albuminuria and advanced kidney disease.

## Materials and methods

### Study patients

All patients with type 2 DM who visited the diabetes outpatient clinics of two hospitals in southern Taiwan were included in this study. The exclusion criteria were patients: (1) with type 1 DM (defined as those who presented with acute hyperglycemic symptoms with high ketonuria [≥3], diabetic ketoacidosis, or the continuous use of insulin for the year after the diagnosis with low C peptide level, with positive or negative of glutamic acid decarboxylase autoantibodies upon diagnosis, and issued with catastrophic card by Taiwan National Health Insurance); (2) receiving dialysis; and (3) who had received a renal transplantation. The study protocol was approved by the Institutional Review Board of Kaohsiung Medical University Hospital (KMUHIRB-E-20150029), and all methods were carried out in accordance with the approved guidelines. In addition, all of the participants provided written informed consent to participate in this study.

### Collection of demographic, medical, and laboratory data

Data on age, sex, and comorbidities were obtained from medical records and interviews with the patients. Fasting blood samples were obtained from all of the patients and analyzed using a COBAS Integra 400autoanalyzer (Roche Diagnostics GmbH, D-68298 Mannheim, Germany). Serum creatinine was measured using the compensated Jaffé method as previously reported [21], and estimated GFR (eGFR) values were calculated using the Chronic Kidney Disease Epidemiology Collaboration equation (CKD-EPI eGFR) [22]. In addition, data on fasting glucose, glycated hemoglobin (HbA_1c_), triglycerides [TGs], total cholesterol, high-density lipoprotein [HDL]-cholesterol, and low-density lipoprotein [LDL]-cholesterol were also recorded. Data on the use of medications including oral anti-diabetic agents, insulin, angiotensin converting enzyme inhibitors (ACEIs) and/or angiotensin II receptor blockers (ARBs), statins, and fibrate during the study period were obtained from medical records.

### Definition of coronary artery disease and cerebrovascular disease

Coronary artery disease was defined as a history of angina, ischemic changes on electrocardiography, old myocardial infarction, or having received coronary bypass surgery or angioplasty. Cerebrovascular disease was defined as a history of cerebrovascular events including infarction and cerebral bleeding.

### Definition of albuminuria and advanced kidney disease

Urine levels of albumin and creatinine were measured from spot urine samples using a COBAS Integra 400 plus autoanalyzer (Roche Diagnostics, Indianapolis, IN). Albuminuria was defined as a urine albumin/creatinine ratio of ≥30 mg/g. Patients with evidence of kidney damage for >3 months and an eGFR of 30–59 mL/min/1.73 m^2^, 15–29 mL/min/1.73 m^2^, and <15 mL/min/1.73 m^2^ were classified as having CKD stages 3, 4 and 5, respectively. Advanced kidney disease was defined as an eGFR <30 mL/min/1.73 m^2^ in this study.

### Calculation of obesity-related indices

1. BMI was calculated as:

$$\mathrm{BMI}=\text{body weight }(\mathrm{BW})\;(\mathrm{kg})/\text{body height }{\left(\mathrm{BH}\right)}^{2}\;(m)$$

2. WHtR was calculated as:

WHtR=waist circumference (WC) (cm)/ BH (cm)

3. WHR was calculated as:

WHR=WC (cm)/ hip circumference (HC) (cm)

4. LAP was calculated as:

$$\mathrm{LAP}=({\mathrm{WC}}_{\left(\mathrm{cm}\right)}-65)\times \;{\mathrm{TG}}_{\left(\mathrm{mmol}/L\right)}\text{ in males},\;\mathrm{and}$$

$$\mathrm{LAP}=({\mathrm{WC}}_{\left(\mathrm{cm}\right)}-58)\times \;{\mathrm{TG}}_{\left(\mathrm{mmol}/L\right)}\text{ in }\mathrm{females}.$$

[8]

5. BRI was calculated as:

$$\mathrm{BRI}=364.2-365.5\;\times \sqrt{1-{\left(\frac{\frac{{\mathrm{WC}}_{\left(m\right)}}{2\pi }}{0.5\;\times {\mathrm{BH}}_{\left(m\right)}}\right)}^{2}\;}.$$

[9]

6. CI was calculated using the Valdez equation based on BW, BH, and WC as:

$$\mathrm{CI}=\frac{{\mathrm{WC}}_{\left(m\right)}}{0.109\;\times \sqrt{\frac{{\mathrm{BW}}_{\left(\mathrm{kg}\right)}}{{\mathrm{BH}}_{\left(m\right)}}}\;}.$$

[10]

7. VAI score was calculated as described previously [11] using the following sex-specific equations (with TG levels in mmol/l and HDL-cholesterol levels in mmol/l):

$$\mathrm{VAI}=(\frac{{\mathrm{WC}}_{\left(\mathrm{cm}\right)}}{39.68\;+(1.88\;\times \;\mathrm{BMI})})\times (\frac{{\mathrm{TG}}_{\left(\mathrm{mmol}/L\right)}}{1.03})\times (\frac{1.31}{{\mathrm{HDL}}_{(\mathrm{mmol}/L)}})\text{in males},\;\mathrm{and}$$

$$\mathrm{VAI}=(\frac{{\mathrm{WC}}_{\left(\mathrm{cm}\right)}}{36.58\;+(1.89\;\times \;\mathrm{BMI})})\times (\frac{{\mathrm{TG}}_{\left(\mathrm{mmol}/L\right)}}{0.81})\times (\frac{1.52}{{\mathrm{HDL}}_{\left(\mathrm{mmol}/L\right)}})\text{in females}.$$

8. BAI was calculated according to the method of Bergman and colleagues as:

$$\mathrm{BAI}=\frac{{\mathrm{HC}}_{\left(\mathrm{cm}\right)}}{{{\mathrm{BH}}_{\left(m\right)}}^{3/2}}\;-18.$$

[12]

9. AVI was calculated as

$$\mathrm{AVI}=\frac{2\;\times {\;({\mathrm{WC}}_{\left(\mathrm{cm}\right)})\;}^{2}+0.7\;\times {\;({\mathrm{WC}}_{\left(\mathrm{cm}\right)}-{\text{HC }}_{\left(\mathrm{cm}\right)})}^{2}}{1000}.$$

[13]

10. ABSI was calculated as:

$$\mathrm{ABSI}=\mathrm{WC}\;(m)/\left[{\mathrm{BMI}}^{2/3}(\mathrm{kg}/m^{2})\times {\mathrm{BH}}^{1/2}(m)\right].$$

[14]

11. TyG index was calculated as

TyG index=Ln [fasting TG (mg/dL)×fasting plasma glucose (mg/dL)/2].

[15]

### Statistical analysis

Data are presented as percentage, mean ± standard deviation, or median (25th–75th percentile) for TGs. Characteristics of the study patients for the continuous and categorical variables were analyzed by *t* test/Wilcoxon Rank Sum test and the Chi-squared test/Fisher exact test, as appropriate, for comparisons between groups. Multiple logistic regression analysis was used to identify the factors associated with albuminuria and advanced kidney disease. There were four different sets of adjustments depending on which obesity index is being studied to avoid repeated adjustments in multivariable model. Different multivariable logistic regression analyses were performed for different indices as follows:

1. Adjusted for age, sex, cerebrovascular disease, coronary artery disease, systolic and diastolic blood pressures, fasting glucose, HbA_1c_, log TGs, total cholesterol, HDL-cholesterol, LDL-cholesterol, eGFR, and use of medications for BMI, WHR, WHtR, BRI, CI, BAI, AVI, and ABSI.
2. Adjusted for age, sex, cerebrovascular disease, coronary artery disease, systolic and diastolic blood pressures, fasting glucose, HbA_1c_, total cholesterol, HDL-cholesterol, LDL-cholesterol, and the use of medications for LAP.
3. Adjusted for age, sex, cerebrovascular disease, coronary artery disease, systolic and diastolic blood pressures, fasting glucose, HbA_1c_, total cholesterol, LDL-cholesterol, and the use of medications for VAI.
4. Adjusted for age, sex, cerebrovascular disease, coronary artery disease, systolic and diastolic blood pressures, HbA_1c_, total cholesterol, HDL-cholesterol, LDL-cholesterol, and the use of medications for TyG index.

A difference was considered significant if the *p* value was <0.05. All statistical analyses were performed using SPSS version 19.0 for Windows (SPSS Inc. Chicago, IL).

## Results

A total of 1872 patients (mean age 64.0 ± 11.3 years, 809 males and 1063 females) were included in this study, of whom 34.7% had albuminuria and 3.0% had advanced kidney disease.

### Comparison of baseline characteristics between the patients with and without albuminuria

Comparisons of the baseline characteristics between the patients with and without albuminuria are shown in Table 1. Compared to the patients without albuminuria, those with albuminuria were older, predominantly female, and had higher rates of coronary artery disease and cerebrovascular disease. In addition, they had higher systolic and diastolic blood pressures, higher BW, higher WC, higher HC, higher levels of fasting glucose, HbA_1c_, TGs, total cholesterol, and a lower eGFR and lower level of HDL-cholesterol. They also had higher rates of insulin, ACEI and/or ARB and fibrate use. Moreover, the patients with albuminuria had higher values of all of the obesity-related indices (BMI, WHR, WHtR, LAP, BRI, CI, VAI, BAI, AVI, ABSI, and TyG index).

**Table 1.** Comparison of baseline characteristics between patients with and without albuminuria.

Characteristics	Without albuminuria (*n* = 1,223)	With albuminuria (*n* = 649)	*p*
Age (year)	63.6 ± 11.3	64.7 ± 11.2	0.023
Male gender (%)	45.1	39.4	0.018
Coronary artery disease (%)	15.2	20.3	0.005
Cerebrovascular disease (%)	4.1	7.1	0.005
Systolic blood pressure (mmHg)	132.6 ± 17.5	139.3 ± 20.4	<0.001
Diastolic blood pressure (mmHg)	77.0 ± 10.7	79.5 ± 12.0	<0.001
Body height (cm)	159.2 ± 8.2	158.6 ± 8.6	0.111
Body weight (kg)	64.9 ± 10.6	66.8 ± 11.4	0.001
Waist circumference (cm)	88.6 ± 9.2	91.6 ± 9.9	<0.001
Hip circumference (cm)	97.9 ± 7.1	99.7 ± 8.5	<0.001
BMI category (%)			<0.001
Underweight (BMI <18.5 kg/m^2^)	0.7	0.8	
Normal (18.5 ≤ BMI <24 kg/m^2^)	33.4	24.7	
Overweight (24 ≤ BMI <27 kg/m^2^)	36.0	31.9	
Mild obesity (27 ≤ BMI <30 kg/m^2^)	19.3	25.7	
Moderate obesity (30 ≤ BMI <35 kg/m^2^)	9.5	51.1	
Severe obesity (BMI ≥35 kg/m^2^)	1.1	1.8	
Laboratory parameters			
Fasting glucose (mg/dL)	144.6 ± 50.3	156.1 ± 54.3	<0.001
HbA_1c_ (%)	7.5 ± 1.6	8.0 ± 1.8	<0.001
Triglyceride (mg/dL)	117 (86-165)	141 (101.5-204)	<0.001
Total cholesterol (mg/dL)	183.1 ± 35.3	190.4 ± 43.2	0.002
HDL-cholesterol (mg/dL)	50.5 ± 13.2	48.2 ± 12.7	<0.001
LDL-cholesterol (mg/dL)	103.4 ± 26.9	106.2 ± 30.6	0.145
eGFR (mL/min/1.73 m^2^)	69.9 ± 19.0	65.1 ± 22.6	<0.001
Medications			
Oral anti-diabetic drugs (%)	90.5	89.3	0.433
Insulin (%)	35.7	48.5	<0.001
ACEI and/or ARB (%)	67.3	85.2	<0.001
Statins use (%)	58.3	62.0	0.123
Fibrate use (%)	14.0	20.9	<0.001
Obesity related indices			
BMI (kg/m^2^)	25.5 ± 3.4	26.5 ± 3.7	<0.001
WHR	0.91 ± 0.06	0.92 ± 0.07	<0.001
WHtR	0.56 ± 0.06	0.58 ± 0.06	<0.001
LAP	44.1 ± 32.3	62.6 ± 56.7	<0.001
BRI	4.6 ± 1.3	5.0 ± 1.4	<0.001
CI	1.28 ± 0.08	1.30 ± 0.09	<0.001
VAI	2.3 ± 2.4	3.1 ± 3.4	<0.001
BAI	31.0 ± 4.9	32.1 ± 5.5	<0.001
AVI	16.0 ± 3.3	17.0 ± 3.7	<0.001
ABSI	0.081 ± 0.005	0.082 ± 0.006	<0.001
TyG index	9.0 ± 0.6	9.3 ± 0.7	<0.001

BMI: body mass index; HbA_1c_: glycated hemoglobin; HDL: high-density lipoprotein; LDL: low-density lipoprotein; eGFR: estimated glomerular filtration rate; ACEI: angiotensin converting enzyme inhibitor; ARB: angiotensin II receptor blocker; WHR: waist–hip ratio; WHtR: waist-to-height ratio; LAP: lipid accumulation product; BRI: body roundness index; CI: conicity index; VAI: visceral adiposity index; BAI: body adiposity index; AVI: abdominal volume index; ABSI: a body shape index; TyG index: triglyceride glucose index.

### Determinants of albuminuria

Table 2 shows the determinants of albuminuria in the study patients as determined in multivariable logistic regression analysis. After multivariable logistic regression analysis, high BMI (*p* = 0.003), high WHR (*p* < 0.001), high LAP (*p* < 0.001), high BRI (*p* < 0.001), high CI (*p* < 0.001), high VAI (*p* = 0.006), high BAI (*p* = 0.006), high AVI (*p* < 0.001), high ABSI (*p* = 0.011), and high TyG index (*p* = 0.032) were significantly associated with albuminuria.

**Table 2.** Association of obesity related indices with albuminuria using multivariable logistic regression analysis.

Obesity-related indices	Multivariable	
	OR (95% CI)	*P*
BMI (per 1 kg/m^2^)^a^	1.047 (1.016–1.078)	0.003
WHR (per 0.1)^a^	1.224 (1.031–1.453)	0.021
WHtR (per 0.1)^a^	1.429 (1.193–1.712)	<0.001
LAP (per 1)^b^	1.008 (1.004–1.013)	<0.001
BRI (per 1)^a^	1.177 (1.085–1.277)	<0.001
CI (per 0.1)^a^	1.274 (1.114–1.456)	<0.001
VAI (per 1)^c^	1.074 (1.020–1.130)	0.006
BAI (per 1)^a^	1.034 (1.010–1.059)	0.006
AVI (per 1)^a^	1.075 (1.041–1.110)	<0.001
ABSI (per 0.01)^a^	1.316 (1.064–1.627)	0.011
TyG index (per 1)^d^	1.307 (1.024–1.668)	0.032

Values expressed as odds ratio (OR) and 95% confidence interval (CI). Abbreviations are the same as in Table 1.

^a^Adjusted for age, sex, coronary artery disease, cerebrovascular disease, systolic and diastolic blood pressures, fasting glucose, HbA_1c_, log triglyceride, total cholesterol, HDL-cholesterol, LDL-cholesterol, eGFR, and medications use.

^b^Adjusted for age, sex, coronary artery disease, cerebrovascular disease, systolic and diastolic blood pressures, fasting glucose, HbA_1c_, total cholesterol, HDL-cholesterol, LDL-cholesterol, eGFR, and medications use.

^c^Adjusted for age, sex, coronary artery disease, cerebrovascular disease, systolic and diastolic blood pressures, fasting glucose, HbA_1c_, total cholesterol, LDL-cholesterol, eGFR, and medications use.

^d^Adjusted for age, sex, coronary artery disease, cerebrovascular disease, systolic and diastolic blood pressures, HbA_1c_, total cholesterol, HDL-cholesterol, LDL-cholesterol, eGFR, and medications use.

### Comparison of baseline characteristics between the patients with and without eGFR < 30 mL/min/1.73m^2^

Comparisons of the baseline characteristics between the patients with and without eGFR <30 mL/min/1.73 m^2^ are shown in Table 3. Compared to the patients with eGFR ≥30 mL/min/1.73m^2^, those with eGFR <30 mL/min/1.73 m^2^ were older, had higher rates of coronary artery disease and cerebrovascular disease. In addition, they had higher systolic blood pressure, higher BW, higher WC, higher HC, lower fasting glucose, lower HbA_1c_, higher TG level, lower HDL-cholesterol, and lower eGFR. They also had a lower rate of oral anti-diabetic drug use, and higher rates of insulin, ACEI and/or ARB, statin and fibrate use. Moreover, the patients with eGFR < 30 mL/min/1.73 m^2^ had higher values of all of the obesity-related indices (BMI, WHR, WHtR, LAP, BRI, CI, VAI, BAI, AVI, and ABSI) except TyG index.

**Table 3.** Comparison of baseline characteristics between patients with and without eGFR <30 mL/min/1.73 m^2^.

Characteristics	eGF*R* ≥ 30 (*n* = 1,815)	eGF*R* < 30 (*n* = 57)	*P*
Age (year)	63.8 ± 11.2	72.6 ± 10.2	<0.001
Male gender (%)	43.3	40.4	0.681
Coronary artery disease (%)	16.4	36.5	<0.001
Cerebrovascular disease (%)	5.0	11.5	0.034
Systolic blood pressure (mmHg)	134.8 ± 18.7	140.3 ± 21.1	0.031
Diastolic blood pressure (mmHg)	78.0 ± 11.2	74.6 ± 11.9	0.071
Body height (cm)	159.0 ± 8.4	157.6 ± 8.7	0.262
Body weight (Kg)	65.4 ± 11.0	69.2 ± 9.9	0.008
Waist circumference (cm)	89.4 ± 9.5	96.6 ± 8.5	<0.001
Hip circumference (cm)	98.4 ± 7.7	101.5 ± 6.8	0.001
BMI category (%)			0.001
Underweight (BMI <18.5 kg/m^2^)	0.7	0	
Normal (18.5 ≤ BMI <24 kg/m^2^)	31.1	7.7	
Overweight (24 ≤ BMI <27 kg/m^2^)	34.5	36.5	
Mild obesity (27 ≤ BMI <30 kg/m^2^)	21.4	26.9	
Moderate obesity (30 ≤ BMI <35 kg/m^2^)	11.0	26.9	
Severe obesity (BMI ≥ 35 kg/m^2^)	1.4	1.9	
Laboratory parameters			
Fasting glucose (mg/dL)	149.0 ± 51.9	132.9 ± 49.8	0.006
HbA_1c_ (%)	7.7 ± 1.7	7.2 ± 1.2	0.018
Triglyceride (mg/dL)	124 (90–175)	170 (127.25–223.5)	<0.001
Total cholesterol (mg/dL)	185.6 ± 38.2	185.1 ± 41.7	0.792
HDL-cholesterol (mg/dL)	49.8 ± 13.1	44.9 ± 12.4	0.014
LDL-cholesterol (mg/dL)	104.4 ± 28.2	103.5 ± 28.4	0.786
eGFR (mL/min/1.73 m^2^)	69.5 ± 19.2	23.1 ± 5.0	<0.001
Medications			
Oral anti-diabetic drugs (%)	90.3	80.8	0.023
Insulin (%)	39.2	76.9	<0.001
ACEI and/or ARB (%)	72.8	98.1	<0.001
Statins use (%)	59.1	73.1	0.043
Fibrate use (%)	16.0	28.8	0.013
Obesity related indices			
BMI (kg/m^2^)	25.8 ± 3.6	27.8 ± 3.1	<0.001
WHR	0.91 ± 0.07	0.95 ± 0.07	<0.001
WHtR	0.56 ± 0.06	0.61 ± 0.06	<0.001
LAP	49.6 ± 41.8	82.5 ± 74.0	<0.001
BRI	4.7 ± 1.3	5.8 ± 1.4	<0.001
CI	1.28 ± 0.08	1.34 ± 0.08	<0.001
VAI	2.5 ± 2.8	4.0 ± 3.9	<0.001
BAI	31.3 ± 5.2	33.6 ± 5.5	0.002
AVI	16.3 ± 3.4	18.8 ± 3.2	<0.001
ABSI	0.081 ± 0.005	0.084 ± 0.005	<0.001
TyG index	9.1 ± 0.7	9.3 ± 0.7	0.156

Abbreviations are the same as in Table 1.

### Determinants of eGFR < 30 mL/min/1.73 m^2^

Table 4 shows the determinants of eGFR <30 mL/min/1.73 m^2^ in the study patients as determined in multivariable logistic regression analysis. After multivariable logistic regression analysis, high BMI (*p* = 0.016), high WHR (*p* = 0.007), high WHtR (*p* = 0.004), high LAP (*p* = 0.001), high BRI (*p* = 0.003), high CI (*p* = 0.034), high VAI (*p* = 0.002), and high AVI (*p* = 0.004) were significantly associated with eGFR < 30 mL/min/1.73 m^2^, whereas BAI, ABSI, and TyG index were not.

**Table 4.** Association of obesity related indices with eGFR <30 mL/min/1.73 m^2^ using multivariable logistic regression analysis.

Obesity-related indices	Multivariable	
	OR (95% CI)	*P*
BMI (per 1 kg/m^2^)^a^	1.112 (1.021–1.211)	0.015
WHR (per 0.1)^a^	1.887 (1.183–3.010)	0.008
WHtR (per 0.1)^a^	2.091 (1.259–3.474)	0.004
LAP (per 1)^b^	1.016 (1.007–1.025)	0.001
BRI (per 1)^a^	1.369 (1.107–1.694)	0.004
CI (per 0.1)^a^	1.501 (1.022–2.206)	0.038
VAI (per 1)^c^	1.137 (1.046–1.236)	0.003
BAI (per 1)^a^	1.041 (0.975–1.111)	0.226
AVI (per 1)^a^	1.137 (1.042–1.242)	0.004
ABSI (per 0.01)^a^	1.445 (0.783–2.666)	0.238
TyG index (per 1)^d^	1.092 (0.600–1.986)	0.774

Values expressed as odds ratio (OR) and 95% confidence interval (CI). Abbreviations are the same as in Table 1.

^a^Adjusted for age, sex, coronary artery disease, cerebrovascular disease, systolic and diastolic blood pressures, fasting glucose, HbA_1c_, log triglyceride, total cholesterol, HDL-cholesterol, LDL-cholesterol, and medications use.

^b^Adjusted for age, sex, coronary artery disease, cerebrovascular disease, systolic and diastolic blood pressures, fasting glucose, HbA_1c_, total cholesterol, HDL-cholesterol, LDL-cholesterol, and medications use.

^c^Adjusted for age, sex, coronary artery disease, cerebrovascular disease, systolic and diastolic blood pressures, fasting glucose, HbA_1c_, total cholesterol, LDL-cholesterol, and medications use.

^d^Adjusted for age, sex, coronary artery disease, cerebrovascular disease, systolic and diastolic blood pressures, HbA_1c_, total cholesterol, HDL-cholesterol, LDL-cholesterol, and medications use.

## Discussion

In this study, we enrolled patients with type 2 DM, and found that all of the studied obesity-related indices (BMI, WHR, WHtR, LAP, BRI, CI, VAI, BAI, AVI, ABSI, and TyG index) were associated with albuminuria (defined as a urine albumin/creatinine ratio of ≥ 30 mg/g) in the patients. In addition, BMI, WHR, WHtR, LAP, BRI, CI, VAI, and AVI were significantly associated with advanced kidney disease (defined as eGFR < 30 mL/min/1.73 m^2^) in the enrolled patients.

The first important finding of this study is that all of the measured obesity-related indices (BMI, WHR, WHtR, LAP, BRI, CI, VAI, BAI, AVI, ABSI, and TyG index) were significantly associated with albuminuria in the enrolled patients. The Prevention of Renal and Vascular End-stage Disease (PREVEND) study conducted in the Netherlands and published in 2003 reported a prevalence of microalbuminuria of 21% or 13% depending on central or peripheral obesity patterns [23]. Approximately half of the patients in the PREVEND study had hypertension; however, the prevalence of albuminuria in the patients with and without hypertension was not provided. Data from a United Kingdom screening program in 2009 showed that the incidence of microalbuminuria increased with increasing BMI, and that this association was even more significant in patients with a higher BMI [24]. In Ireland, Martin et al. [6] also revealed a significant positive correlation between increasing BMI and increasing proteinuria amongst people with obesity and CKD, and this relationship was even stronger in males and patients with CKD stages 4 and 5. Previous studies have shown that LAP is an effective marker for identifying metabolic obesity and that it is associated with metabolic syndrome [25,26]. In addition, Dai et al. [27] reported that VAI and LAP had superior predictive ability for identifying CKD compared to traditional indices in women. LAP has the advantages of being low cost and easy to measure, and it may be a simpler and more sensitive predictor of albuminuria than traditional indices. Besides, many studies had shown that a higher TyG index is associated with a higher risk of microalbuminuria and chronic kidney disease, which may be due to changes in renal endothelial function and hemodynamics caused by the reaction of insulin resistance [28,29]. Several studies on genetically obese rats and force-fed dogs reported the early onset of changes in hemodynamic sand kidney function characterized by increases in effective plasma blood flow and GFR along with variable increases in albumin excretion and filtration fraction [30,31]. A possible mechanism for these findings is the secretion of inflammatory factors from central or visceral adipose tissue such as tumor necrosis factor-alpha and interleukin-6 which causes endothelial dysfunction at the glomerular level, resulting in an increase in urine albumin excretion [32,33]. This highlights the important role that visceral fat plays in the development of albuminuria, and also that evaluating obesity-related indices may be useful as possible indicators of albuminuria.

The second important finding of this study is that BMI, WHR, WHtR, LAP, BRI, CI, VAI, and AVI were significantly associated with advanced kidney disease in the enrolled patients. Munkhaugen et al. [34] evaluated 75,000 volunteers in a cohort study conducted over a 20-year period in Norway, and found a strong association between BMI and the risk of kidney disease. In another large population-based case-controlled study reported by Ejerblad et al., a BMI of >25 kg/m^2^ at 20 years of age was associated with a three-fold higher risk of developing new-onset kidney disease, even after correcting for hypertension and diabetes. Moreover, the coexistence of diabetes and obesity in their study was associated with a two-fold higher risk of new-onset kidney disease [35]. Cao et al. [36] evaluated 666 elderly population living in the Chinese community with a baseline eGFR ≥60 mL/min/1.73 m^2^ and negative microalbuminuria completing a 3-year follow-up. They found TG ≥1.7 mmol/L increased the risk of eGFR <60 mL/min/1.73 m^2^ by 1.44-fold, of microalbuminuria by 32%, and of developing both abnormality by 1.41-fold. Besides, WC (≥90 cm in men and ≥85 cm in women) were associated with a 1.68-fold higher risk of eGFR <60 mL/min/1.73 m^2^ and a 1.43-fold risk of microalbuminuria and a 1.89-fold risk of developing both abnormality. High TG levels and central obesity are risk factors for kidney injury in 3 years. The exact mechanisms by which obesity may worsen or cause CKD remain unclear. However, it is possible that excess fat mass increases the risks of type 2 DM, hypertension, and atherosclerosis, thereby indirectly leading to CKD [37,38]. Several randomized controlled trials and large observational studies had shown that metabolic surgery and drug therapy significantly improve the control of type 2 diabetes, and this metabolic benefit could improve long-term renal prognosis [39,40]. Evidence that obesity treatment could improve renal outcomes in patients with type 2 diabetes supported the pathogenic role of obesity in CKD. Obesity may also directly affect the kidneys pathophysiologically through alterations in the inflammatory milieu and renal hemodynamics, and the production of adipokines and growth factors [38,41]. These effects include oxidative stress, inflammation, activation of the renin–angiotensin–aldosterone system, abnormal lipid metabolism, increased production of insulin, and insulin resistance [42,43]. These effects can then result in the ectopic accumulation of lipids and increases in renal sinus fat, glomerular hypertension, and increased permeability of the glomeruli through glomerular filtration barrier injury related to hyperfiltration, and consequently glomerulomegaly and focal or segmental glomerulosclerosis [44,45]. This mechanism could also explain that the higher VAI was associated with incident CKD, and many previous studies had also proved the same result [46,47]. In previous meta-analyses and systemic reviews, WHtR was shown to have the best discriminatory ability for the risk of hypertension, diabetes, and dyslipidemia [48,49]. WHtR may be a simpler and more predictive indicator of the cardiometabolic risk factors associated with central obesity than other anthropometric indices. Thomas et al. [9] developed the BRI as an index to predict the percentage of visceral adipose tissue and body fat to provide an initial overview of a person’s physical health. Recent studies have shown that the BRI can be used as an indicator of adipose tissue to detect the presence of CVD, left ventricular hypertrophy, diabetes, and hyperuricemia [50,51]. Taken together, these easy to measure indicators may be suitable as tools to screen for the risk of CKD.

There are several limitations to this study. First, the study included patients with type 2 DM regardless of its duration, and the duration of DM can affect the inhomogeneity of the effects of diabetes on nephropathy. Second, the diagnosis of albuminuria was based on a single laboratory measurement. However, this limitation was minimized by the accuracy of the urinary albumin and creatinine assays as described in the Methods section. Third, data on some important variables that can influence diabetic nephropathy, such as smoking history, albumin, C-Reactive protein, and CKD etiology, were lacking. Finally, this study was cross-sectional in design, and so causal relationships and long-term clinical outcomes could not be confirmed. Nonetheless, the results may help to shed light on the importance of obesity-related indices on diabetic nephropathy in this population. Further prospective studies are needed to evaluate the development and progression of diabetic nephropathy in patients with diabetes.

In conclusion, the results of this study showed that various obesity-related indices were significantly associated with albuminuria and advanced kidney disease in patients with type 2 DM. It is, therefore, important to reduce obesity through lifestyle changes and regular exercise. Screening may be considered in public health programs to recognize and take appropriate steps to prevent subsequent complications.
